# Complete Right Upper Lobe Atelectasis Caused by an Endobronchial Hamartoma: Diagnostic Challenges and Successful Treatment With Rigid Bronchoscopy and Laser

**DOI:** 10.1002/rcr2.70638

**Published:** 2026-05-27

**Authors:** Meridiana Dodaj, Lorenzo Carriera, Giovanni Marchetti, Stefano Baglioni, Roberto Lipsi

**Affiliations:** ^1^ Department of Pulmonology and Sub‐Intensive Respiratory Unit Ospedale Santa Maria Della Misericordia Perugia Italy; ^2^ Facoltà di Medicina e Chirurgia Università Cattolica Del Sacro Cuore Rome Italy; ^3^ Division of Pathology Ospedale Santa Maria Della Misericordia Perugia Italy

**Keywords:** airway obstruction, atelectasis, endobronchial hamartoma, laser resection, rigid bronchoscopy

## Abstract

Endobronchial hamartomas are very rare benign lung tumours that may cause airway obstruction and are frequently misdiagnosed due to limited biopsy samples. A 53‐year‐old overweight male, heavy smoker with hypertension, presented to the emergency department with dyspnoea and cough not responsive to antibiotics and steroid therapy. Laboratory tests, including inflammatory markers, were normal. Chest X‐ray and computed tomography showed complete atelectasis of the right upper lobe while flexible bronchoscopy revealed an obstructive endobronchial lesion. Initial bronchoscopic biopsies suggested a lipoma while definitive diagnosis of endobronchial hamartoma was achieved after obtaining larger tissue samples during rigid bronchoscopy. The lesion was completely removed using a coring‐out technique with rigid bronchoscopy and laser coagulation, leading to full airway recanalization and clinical recovery. Endobronchial hamartomas may mimic other benign tumours on limited biopsy specimens. Rigid bronchoscopy allows both definitive diagnosis and effective treatment, preventing unnecessary surgical resection.

## Graphical abstract

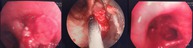

## 1 Introduction

Pulmonary hamartomas are the most common benign tumours of the lung [1] and are typically located in the peripheral lung parenchyma. In contrast, endobronchial hamartomas are rare, accounting for less than 10% of all pulmonary hamartomas [2]. Flexible bronchoscopy is usually the first diagnostic approach; however, biopsy samples obtained through this technique may be limited and insufficient to establish a definitive diagnosis. In particular, sampling of a single tissue component, such as adipose tissue, may result in misdiagnosis, most commonly as endobronchial lipoma [2]. Rigid bronchoscopy plays a pivotal role in the diagnosis and management of endobronchial hamartomas, as it allows the acquisition of larger and more representative tissue samples for definitive histopathological evaluation. Moreover, it enables complete endoscopic resection through mechanical debulking, often combined with adjunctive techniques such as laser therapy or electrocautery [3, 4]. Early diagnosis and bronchoscopic treatment are crucial to prevent irreversible suppurative lung damage secondary to recurrent infections, which may ultimately require surgical resection. In this report, we describe a case of complete right upper lobe atelectasis caused by an endobronchial hamartoma that was initially misdiagnosed on flexible bronchoscopic biopsy and was successfully treated with rigid bronchoscopy and laser therapy.

## 2 Case Report

**FIGURE 1:**
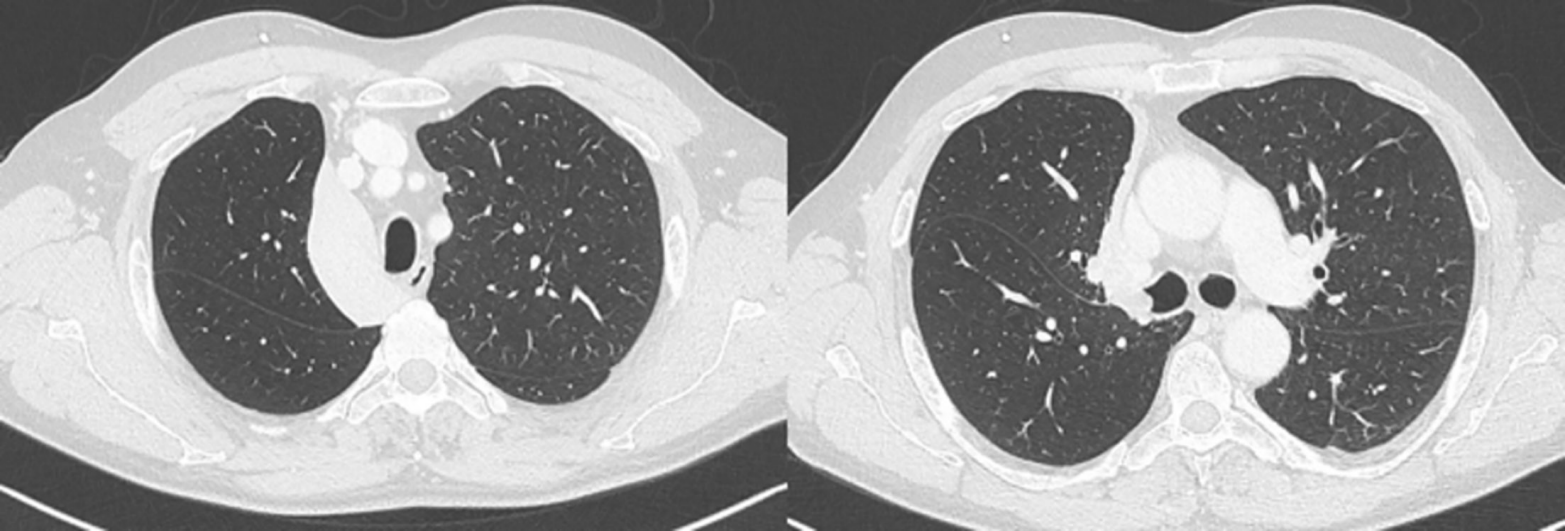
Chest CT scan performed at admission. Chest CT at admission shows right upper lobe atelectasis (left panel) with an endobronchial obstructing lesion in the right upper lobe bronchus (right panel).

**FIGURE 2:**
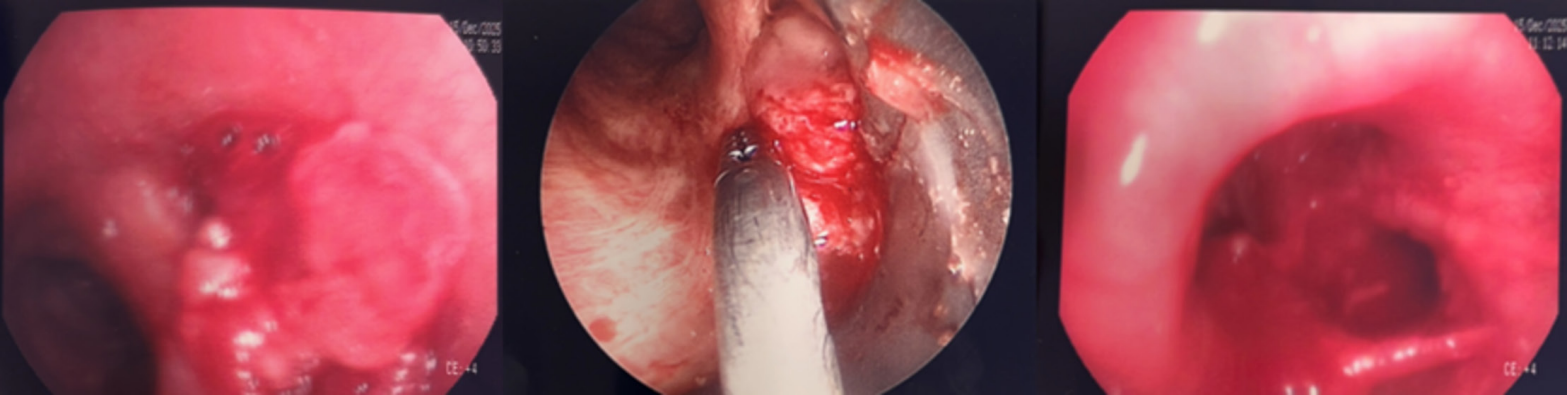
Sequential bronchoscopic appearance of the right upper lobe bronchus before, during, and after endoscopic treatment. Images obtained during rigid bronchoscopy. Left: Complete obstruction of the right upper lobe bronchus by an endobronchial lesion before treatment. Middle: Intra‐procedural view showing the debulking phase with the instrument in situ. Right: Post‐treatment view demonstrating restoration of airway patency after tumour debulking and laser coagulation.

**FIGURE 3:**
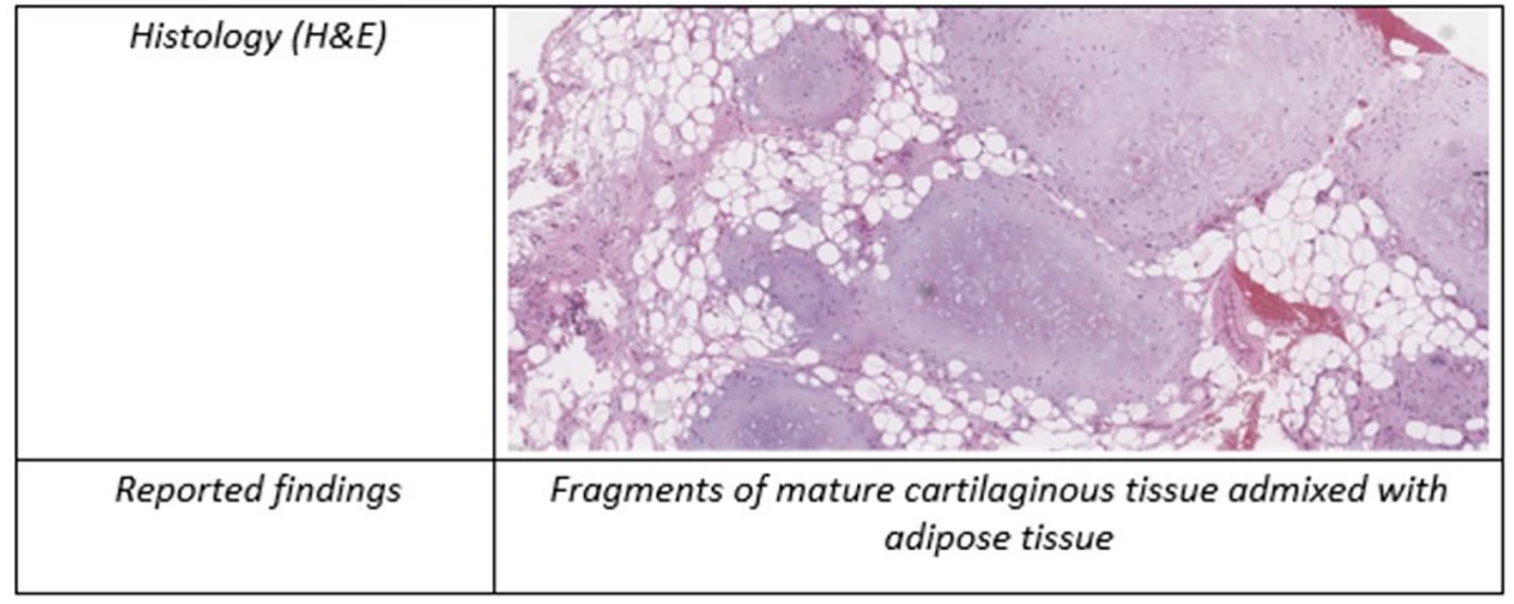
Histopathology of the endobronchial lesion.

**FIGURE 4:**
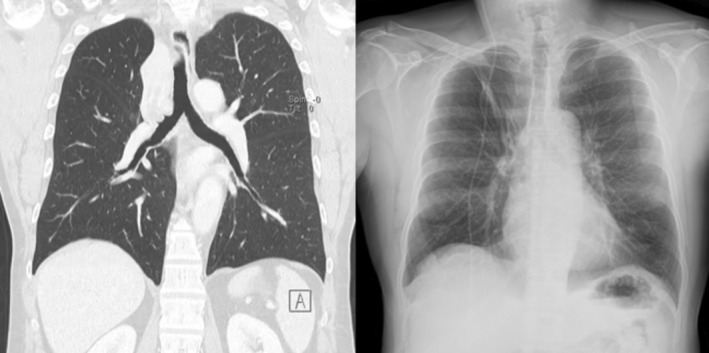
Radiological comparison before and after bronchoscopic treatment. Left: Coronal contrast‐enhanced chest CT demonstrating complete right upper lobe atelectasis due to obstruction of the right upper lobar bronchus. Right: Post‐procedural chest X‐ray demonstrating re‐expansion of the right upper lobe after rigid bronchoscopic debulking.

A 53‐year‐old man, heavy smoker, overweight, with a history of hypertension and no known allergies, presented to the emergency department with progressive dyspnoea and cough, unresponsive to antibiotic therapy and oral corticosteroids. Physical examination showed reduced breath sounds over the right upper lung field. Laboratory investigations, including inflammatory markers, were within normal limits. A contrast‐enhanced chest computed tomography (CT) scan was performed and demonstrated complete right upper lobe atelectasis, with associated volume loss and non‐visualization of the right upper lobar bronchus at its origin (Figure 1). No definite air bronchogram was identified within the collapsed lobe. These findings suggested obstruction of the right upper lobar bronchus, mainly due to an obstructing endobronchial lesion or, less likely, bronchial infiltration. Bronchoscopic evaluation was therefore recommended. Flexible bronchoscopy demonstrated a smooth, well‐circumscribed endobronchial mass causing complete obstruction of the right upper lobe bronchus. Endobronchial biopsies were performed, and histopathological analysis suggested a lipoma. The patient subsequently underwent diagnostic‐therapeutic rigid bronchoscopy. Complete tumour removal was achieved using a coring‐out technique with the rigid bronchoscope, followed by laser coagulation for haemostasis and optimization of resection (Figure 2). Definitive histopathological evaluation established the diagnosis of endobronchial chondroid hamartoma (Figure 3). The procedure was uneventful. Compared with the pre‐procedural CT findings of complete right upper lobe atelectasis, the post‐procedural chest X‐ray demonstrated re‐expansion of the right upper lobe, confirming radiological improvement after bronchoscopic treatment (Figure 4). The patient remained clinically stable, with resolution of respiratory symptoms, and was discharged without complications. At 2‐month follow‐up, chest radiography showed no recurrent abnormalities.

## 3 Discussion

Clinical presentation of endobronchial hamartomas is often nonspecific and depends on the degree and duration of bronchial obstruction. Symptoms such as dyspnoea, cough, wheezing or recurrent infections may mimic more common respiratory conditions including asthma, chronic obstructive pulmonary disease or bronchogenic carcinoma, particularly in smokers [1, 2]. In our case, the patient presented with progressive dyspnoea, cough and complete right upper lobe atelectasis, despite normal laboratory findings and absence of inflammatory markers, highlighting the importance of imaging and endoscopic evaluation in unexplained lobar collapse [1, 2]. Radiological findings are frequently non‐diagnostic. While CT may suggest a benign lesion when fat density or calcifications are present, these features are not always evident, particularly in purely endobronchial lesions [1, 2]. Consequently, bronchoscopic evaluation plays a pivotal role in diagnosis. Flexible bronchoscopy is usually the first‐line approach; however, as demonstrated in this case, small biopsy samples may be insufficient to establish a definitive diagnosis [4, 5]. The initial histological finding of lipoma likely reflected sampling of the adipose component of the lesion, underscoring a well‐known diagnostic pitfall [4, 5]. Hamartomas are characterized by a disorganized mixture of mature tissues normally found in the lung, including cartilage, fat, fibrous tissue and respiratory epithelium [1, 2]. Accurate diagnosis therefore requires adequately sized specimens containing multiple tissue components. Rigid bronchoscopy allows acquisition of larger and more representative biopsy samples and should be considered when initial flexible bronchoscopic biopsies are inconclusive or discordant with clinical and radiological findings, as small samples may not reliably exclude malignancy in obstructive endobronchial lesions [4, 5]. From a therapeutic standpoint, bronchoscopic resection is the treatment of choice for endobronchial hamartomas without extraluminal extension [3, 4, 5]. Several endoscopic techniques have been described, including mechanical debulking, laser therapy, electrocautery, argon plasma coagulation and cryotherapy [3, 4, 5, 6]. Rigid bronchoscopy offers superior airway control, effective mechanical coring‐out of the lesion, and optimal management of bleeding, particularly in centrally located obstructive airway tumours [3, 4, 5]. In the present case, the combination of rigid bronchoscopy and laser coagulation allowed complete tumour removal, immediate airway recanalization and rapid re‐expansion of the atelectatic lobe through a minimally invasive bronchoscopic approach. Early endoscopic management is crucial, as prolonged bronchial obstruction may lead to post‐obstructive pneumonia, bronchiectasis or irreversible parenchymal damage, potentially necessitating surgical resection [4, 5]. Several studies have demonstrated excellent long‐term outcomes following bronchoscopic treatment, with low recurrence rates and preservation of lung function [3, 4, 5]. Surgical intervention should be reserved for cases with extrabronchial extension, uncertain diagnosis or failed endoscopic treatment [5]. This case highlights the diagnostic limitations of flexible bronchoscopy in endobronchial tumours and underscores the pivotal role of rigid bronchoscopy in achieving both definitive diagnosis and curative treatment of endobronchial hamartomas.

## Author Contributions

Conceptualisation: M.D. and R.L. Data collection: M.D. Writing – original draft preparation: M.D., L.C., G.M., S.B., R.L. Writing – review and editing: M.D., L.C., G.M., S.B., R.L. Supervision: S.B. All authors have read and agreed to the published version of the manuscript.

## Funding

The authors have nothing to report.

## Consent

The authors declare that written informed consent was obtained for the publication of this manuscript and accompanying images using the consent form provided by the Journal.

## Conflicts of Interest

The authors declare no conflicts of interest.

## Data Availability

The data that support the findings of this study are available from the corresponding author upon reasonable request.
